# Approaching the Hard-to-Reach in Organized Colorectal Cancer Screening: an Overview of Individual, Provider and System Level Coping Strategies

**DOI:** 10.3934/publichealth.2017.3.289

**Published:** 2017-06-22

**Authors:** Jason Liwen Huang, Yuan Fang, Miaoyin Liang, Shannon TS Li, Simpson KC Ng, Zero SN Hui, Jessica Ching, Harry Haoxiang Wang, Martin Chi Sang Wong

**Affiliations:** 1 JC School of Public Health and Primary Care, Faculty of Medicine, Chinese University of Hong Kong, Hong Kong SAR, China; 2 JC bowel cancer education center, Prince of Wales Hospital, Chinese University of Hong Kong, Hong Kong SAR, China; 3 School of Public Health, Sun Yat-sen University, Guangzhou 510080, Guangdong, China; 4 General Practice and Primary Care, Institute of Health and Wellbeing, University of Glasgow, UK; 5 Institute of Digestive Disease, Faculty of Medicine, Chinese University of Hong Kong; 6 State Key Laboratory of Digestive Disease, Faculty of Medicine, Chinese University of Hong Kong

**Keywords:** colorectal cancer, screening, participation, uptake, hard-to-reach

## Abstract

**Background:**

Despite the proven effectiveness of colorectal cancer (CRC) screening on reduction of CRC mortality, the uptake of CRC screening remains low. Participation rate is one of determinants for the success of organized population-based screening program. This review aims to identify those who are hard-to-reach, and summarize the strategies to increase their screening rate from individual, provider and system levels.

**Methods:**

A systematic search of electronic English databases was conducted on the factors and strategies of uptake in CRC screening for the hard-to-reach population up to May 2017.

**Discussion:**

The coverage rate and participation rate are two indexes to identify the hard-to-reach population in organized CRC screening program. However, the homeless, new immigrants, people with severe mental illness, the jail intimates, and people with characteristics including lower education levels and/or low socioeconomic status, living in rural/remote areas, without insurance, and racial minorities are usually recognized as hard-to-reach populations. For them, organized screening programs offer a better coverage, while novel invitation approaches for eligible individuals and multiple strategies from primary care physicians are still needed to enhance screening rates among subjects who are hard-to-reach. Suggestions implied the effectiveness of interventions at the system level, including linkages to general practice; use of decision making tools; enlisting supports from coalition; and the continuum from screening to diagnosis and treatment.

**Conclusion:**

Organized CRC screening offers a system access to approach the hard-to-reach populations. To increase their uptake, multiple and novel strategies from individual, provider and system levels should be applied. For policymakers, public healthcare providers and community stakeholders, it is a test to tailor their potential needs and increase their participation rates through continuous efforts to eliminate disparities and inequity in CRC screening service.

## 1. Introduction

Screening has been proven as an effective preventive strategy to reduce colorectal cancer (CRC) related mortality in randomized controlled studies [1]. Despite strong evidence on CRC screening, its uptake remains low globally [2]–[5]. Maximum participation in organized, population-based screening programs is crucial to achieve the greatest health benefits [6]. According to a European screening guideline, a minimum uptake of at least 45% for organized screening program is considered acceptable, yet a participation rate of over 65% is recommended [7]. The National Colorectal Cancer Roundtable (NCCRT) initiative “80% by 2018” has committed to having 80% of adults aged 50 years and older regularly screened for CRC by 2018 in United States [8].

The outreach to the hard-to-reach population in healthcare and medical service is not a new idea. Labeled as hidden population, Lambert and Wiebel's definition for them was “those who are disadvantaged and disenfranchised: the homeless and transient, chronically mentally ill, high school drop-outs, criminal offenders, prostitutes, juvenile delinquents, gang members, runaways and other street people” [9]. These socially disadvantaged groups are difficult for healthcare providers, policymakers or researchers to access cost-efficiently in large numbers [10]. Regarding the eligible population in colorectal cancer screening, there is no universal definition for the hard-to-reach. Ideally, the eligible target population of organized screening should be all the population from any ethnic background. Therefore, the “hard-to-reach” refers to the people if their social circumstances, immigrant status, language, culture or lifestyle make them difficult to be accessed. From a broader perspective, they could also be recognized as the under-screened, non-participants or non-attenders in organized CRC screening program. All of these definitions will be applied in the following search strategy when we address the target subjects.

The global trend of organized population-based CRC screening offers an available access to approach this population [11]. To identify and help those who are hard-to-reach is an issue not only to improve the cost-effectiveness of organized programs and control the disease-related burden, but also to lower the healthcare costs and health inequities across all social groups [12]. This review aims to identify those who are hard-to-reach, and summarize the strategies to increase their uptake of CRC screening.

Studies which addressed factors associated with the hard-to-reach in organized or population-based CRC screening programs and interventions to improve their uptake rate were identified using a systematic search of English electronic databases (Embase, Medline, Psyc Info, Cochrane Database of Systematic Reviews [CDSR], Web of Knowledge and Cumulative Index to Nursing and Allied Health Literature [CINHAL]) from their inception to May 2017. Abstracts of all the identified articles were reviewed and full-texts were obtained.

## 2. Identify the hard-to-reach population in CRC screening

Two indexes commonly used to describe the uptake of population-based screening programs were “coverage rate” and “participation rate” [7]. Although the two terms could not give a definite answer about who are hard-to-reach, they could be a useful tool to identify the hard-to-reach population indirectly.

The “coverage rate” of a screening program by invitation is the extent to which the invitations sent out by the screening program within the defined screening interval include the eligible population [7]. The coverage rate gives information about the performance of the program organization on inviting the target population. The following groups of people are only a part of population usually out of the invitation list.

Homeless persons are especially vulnerable and suffer from worse health than domiciled ethnic and minority populations. Although they are at higher risk for cancer, there are many barriers for them to take routine preventive services delivered to all the eligible citizens [13]. In a recent research, four hundred and forty-three domiciled and homeless subjects aged 50 years and older from two major New York City shelter-based clinics were included [14]. After analysis of screening rates, socio-demographic characteristics, and factors associated with homelessness from medical records, the researcher found that domiciled subjects were more likely to be screened than the homeless ones (41.3% *vs.* 19.7%; *P* < 0.001). Although the homeless and the domiciled received equal provider counseling, more homeless people declined screening (*P* < 0.001). The logistic regression showed only housing, provider counseling, and older age were associated with screening, however, gender, race, duration of homelessness, insurance status, substance and alcohol abuse, chronic diseases, and mental health had no association with screening.

Similarly, the jail intimates represent a large unscreened population. In the US, the jail population had disproportionate representation of African Americans and Latinos, who are more vulnerable to CRC than the general public. Nevertheless, few studies about cancer screening have been conducted among these people. Binswanger et al. [15] conducted a cross-sectional study on the frequency, knowledge, and willingness to cancer screening in 133 random samples of county jail inmates. High willingness and screening knowledge indicated that jail could be an appropriate venue to provide cancer screening because inmates were receptive to jail-based screening.

Immigrants are also more likely to have issues related to communicable obstacles such as language barriers. They tend to be under-screened, and have low accessibility to general practice services. For instance, in England, the uptake rates of faecal test-based programs were lower in localities with higher proportions of immigrants from the Indian subcontinent [16]. In addition, another group of people usually missed are those patients with severe mental illness, who are exposed to higher levels of cancer risks and less chance to be screened than the average [17]. All of these people are just a part of the traditional subgroups of the hard-to-reach population.

Thus, the task of reviewing the coverage rate in all the eligible population is an essential step to evaluate the performance of invitation strategy. Otherwise, when we describe the characteristics of those subjects who are less likely or willing to receive CRC screening, those hard-to-reach subjects could have been neglected.

Participation rate is another important index to approach the hard-to-reach. It is defined as the number of people who have been screened, within a defined time frame following an invitation, as a proportion of all covered people [7]. Since most of organized CRC screening programs are fecael occult blood test (FOBT)-based programs [4], the participation rate should be assessed by two steps, FOBT test and colonoscopy test. Usually, in organized programs, colonoscopy test would be referred only for those with positive primary outcomes, so the users of FOBT test would not be recognized as the hard-to-reach population anymore. Targeting on the hard-to-reach, we would focus on the barriers to increase the participation rate of FOBT test, the number of people who take and return the stool test kit as proportion of all invited population.

As early as in 1997, Vernon reviewed the factors associated with participation among different screening strategies [18], including demographic variables, medical history, and health-related behaviors (knowledge, attitudes, and beliefs). In 2010 and 2014, Day and Gupta systematically reviewed the facilitating factors and barriers associated with participation rate in CRC screening program [19],[20]. Generally speaking, people with lower education levels and/or low socioeconomic status; individuals living in rural/remote areas; subjects without insurance, and racial minorities are recognized as hard-to-reach with low participation rate. In 2016, Wools et al. conducted another systematic review on subject-related factors for CRC screening participation, adding younger age and not having a spouse as barriers to CRC screening [21]. For instance, in Australia, the participation rate of Indigenous population in national CRC screening programs was as low as 0.5% [22], while those divorced or separated, never married or widowed had lower odds of adherence with CRC screening guidelines compared with individuals who were married or cohabiting [20],[23],[24]. Inconvenient access to the service network is another barrier. Due to this, residents in remote areas had a lowest participation rate in Australian National bowel cancer screening program across the nation [2]. Recently, emerging evidence indicated an association between obesity and disability with a relatively low uptake of cancer screening. It is a new challenge to public cancer screening to overcome the disparity of obesity and related mobility-disability [25],[26].

Hence, utilizing the above two indexes is the first step to identify the hard-to-reach and modify these specific factors to increase their CRC screening rate in an organized program. Even without organized program, the surveillance on the use of screening service from these common hard-to-reach people would offer the chance to recognize them and narrow the disparities. In regards to the application of new techniques, geographic information system (GIS) in combination with health service and community organizations is an innovative approach to identify areas with the need for interventions. This strategy has helped researchers successfully to locate the South Asians in Ontario, Canada, who were under-screened for breast, cervical and CRC [27].

## 3. Strategies to increase screening rate in hard-to-reach population

Organized screening programs could achieve better coverage of the target population including hard-to-reach or disadvantaged groups [28]. According to the barriers from the hard-to-reach, the coping strategies could be summarized at individual, provider and system levels.

### 3.1. Individual level

It is a challenge for a population-based strategy to carry out tailoring measure to approach the hard-to-reach population. The eligible population with obvious barriers deserves more attention and arrangement. Novel approaches to individuals might be needed to invite them [20]. Take the mailed outreach invitations as an example, on one hand to provide people from all socioeconomic groups with equal access to the screening offer is a viable option to realize screening equity; on the other hand, different invitation strategies via general practitioners or telephone support in a variety of languages also helped with overcoming the socioeconomic inequality [29].

Invitation letters signed by the referring primary care physician (PCP), and reminders for non-attendee are some intervention strategies that could improve acceptance of CRC screening [30]. Other strategies, like directly mailing FOBT kits [31], the use of video testimonials [32], inflatable colon demonstrations [33], mailed outreach invitations [34], and adoption of faith-based settings embedded in health promotion campaign [35],[36], are all reported to be attractive and effective for the hard-to-reach populations.

No matter in organized or opportunistic screening, according to the health belief model, personal health education on CRC screening is useful to raise self-awareness, increase the relevant health literacy, and relieve the unnecessary mental concern. With the complete knowledge on the risks of CRC, the benefit of screening and potential complications, the eligible subjects, especially those with high risk factors would be more likely to have their own rational decisions and less likely to be hard-to-reach after invitation from public healthcare systems or recommendations from their professionals or network members [5],[37].

### 3.2. Provider level

PCPs play an important role in population-based CRC screening programs [38]. For screenees, they are most accessible professionals across the whole cancer continuum, including screening enrollment, early diagnosis, specialist referral, and care during and after treatment for cancer and any comorbidity [39]. For the healthcare system, they stand in the first line of screening service, assessing stool occult blood test, offering screening options for decision making, collaborating with specialists, and taking surveillance at community level. It was reported that PCPs endorsement and recommendation was one of the key determinants to participate screening [37],[40]. The success of population-based CRC screening program largely depends on the PCPs engagement to integrate effective systems and procedures into screening delivery [38].

However, it was not uncommon that knowledge gaps about screening guidelines hindered PCPs from optimal screening delivery even their awareness of CRC screening was high [41]–[43]. Moreover, individual PCP strategies have little effect. A recent population-based survey including 717 PCPs and their 147,834 rostered participants due for CRC screening revealed that only multiple PCP strategies could raise screening participation (HR = 1.27, 95% CI: 1.16–1.39, *P* < 0.0001 for PCPs using 4–5 *vs.* 0–1 strategies). In this study, PCPs' practice-based strategies included use of standard reminder prompts for cancer screening, having a systematic process to generate lists of screenees due for screening, having a special staff member to manage cancer screening, and using at least one out of the two cancer screening reports issued by government during this time period. This provided practice-specific audit and feedback data on enrolled subjects due for screening [44].

Both out-reach and in-reach from the clinic is encouraged to increase the uptake of the under-screened. Out-reach was defined as those in which interventions were initiated outside of a clinic visit and conversely in-reach as interventions were initiated by a clinic visit. Geng et al. systematically reviewed the screening participation rates from 42 randomized controlled trials that aimed to improve screening among underserved populations [45]. They reported that among outreach studies, community outreach-delivered, culturally tailored, language concordant education interventions appeared to be most effective, while among in-reach studies, language concordant health education showed particular promise. Cole et al. [46] proposed barbershop-based or church-based interventions to address the screening colonoscopies disparities among black men aged 50 or older.

### 3.3. System level

Organized screening requires “an explicit policy with defined age categories, method, and interval for screening in a defined target population with a defined implementation and quality assurance structure, and tracking of cancer in the population” [47]. Senore et al. collected the latest evidence comparing the acceptance between organized and opportunistic settings in CRC screening. It indicated that organized programs could achieve an extensive coverage and to enhance equity of access to the hard-to- reach by offering screening tests for all eligible subjects as the target population [48]. In the context of organized CRC screening, the following system components are the basic prerequisites for the uptake rate of organized CRC screening programs:

(1) The link to general practice. The detachment of FOBT from clinical settings was reported to be a reason for non-uptake in the NHS Bowel Cancer Screening Program [49]. Participants described sampling faeces and storing faecal samples as broaching a cultural taboo, and causing embarrassment. It would reduce perceived importance to completion of the test kit at home when compared with sample collection at a proper healthcare setting.

(2) The use of decision making tools. A negative decision was not necessarily associated with subsequent non-participation in screening programs. Avoiding or delaying decision making was sometimes the reason for non-participation. Thus professional endorsement, repeat invitations, reminders and decision making tools are helpful for those non-participants [50]. However, Essink-Bot et al. [51] reported informed decision-making about CRC screening participation was suboptimal among both individuals with low health literacy and those with adequate health literacy. It implied written materials may be insufficient, especially among subjects who attained lower educational levels, since there might be limitations in information processing capabilities.

(3) The supports from coalitions like organizations. The New York Citywide Colon Cancer Control Coalition (C5) reported its public health effort to increase CRC screening rate [52]. The organizers first created a government-organized advisory committee including steering committee, screening guidelines, summit planning, communications, colonoscopy quality and community health centers. The C5 stakeholders are from physicians, hospitals and other healthcare organizations, insurers with extensive NYC membership, professional organizations, health departments, advocacy groups, survivor organizations and patient navigators. Then a citywide coalition was organized; a broad range of stakeholders were engaged; and a structure was established. Additionally, since the inception of the program, they also addressed the health disparity and inequity as a core goal. It targets on Russian- and Chinese- speaking communities, leveraging endoscopy centers to provide free colonoscopy screening. The colonoscopy screening rate of the New York City increased from 42% in 2003 to 62% by 2007 and then to almost 70% in 2014, with racial and ethnic disparities minimized. Based on the database analysis from the National Health Interview Survey and the Area Resource, Stimpson et al. [53] suggested that improving access to care alone would not lead to a promising reduction of racial and ethnic disparities in CRC screening. Rather than individual factors, additional measures are needed to be implemented, including improved cultural competency of providers, patient navigators, and granting workers paid leaves to obtain screening and diagnostic testing.

(4) The continuum from screening to diagnosis and treatment. The hard-to-reach populations in CRC screening are often the groups of people under-severed for diagnostic follow-up, and CRC treatment. This population requires continuous funding support by policies embedded in programs that improved follow-up and treatment of CRC [20].

In 2005, during the initial recruitment stage of a five-site screening program to low-income, uninsured, and underinsured individuals, the funded sites experienced unexpectedly low participation rates. The public health practitioners worked closely with the health care sector to implement comprehensive strategies across individual, provider, and system levels [54]. Efforts included expanding partnerships with additional primary care networks for greater outreach; enhancing support to providers to address organizational and systemic barriers to encourage more consistent engagement; and developing tailored outreach and education for non-participants. The participation rates the program finally doubled in one year after their evidenced-based and continuous effort.

## 4. Conclusion

The identification of hard-to-reach subjects for an organized CRC screening program is the first step to improve coverage rate among the prospective screening participants. Novel approaches in order to enroll these hard-to-reach individuals are needed. The best screening test is the one that gets completed. To increase their uptake of CRC screening is a test for policymakers, healthcare providers and community stakeholders to tailor their potential needs, increase their participation rate, and eliminate disparities and inequity in the program. Future research studies should evaluate various strategies that may improve identification of hard-to-reach individuals. And at the same time, the effectiveness to engage these subjects into the screening programs is also desired for improvement. The persistent compliance rate of screening among the hard-to-reach subjects needs to be examined and independently, whereas associated factors should be explored to inform the tailor-made design of future compliance-enhancing strategies.
